# Supplementary material for: Finite element simulation study on vertical bearing characteristics of single pile with ram-compacted bearing sphere
Source: PLoS One. 2023 Sep 21;18(9):e0291719. doi: 10.1371/journal.pone.0291719 (PMC10513323; doi:10.1371/journal.pone.0291719)
Supplement: S1 Data — (ZIP) [file pone.0291719.s001.zip › The values used to build graphs/Axial force and lateral frictional resistance/Regarding Tables 5 and 6.docx]

The data from ZC1 to ZC6 in Tables 5 and 6 can be found in the corresponding file names in this folder.
