# Supplementary material for: Finite element simulation study on vertical bearing characteristics of single pile with ram-compacted bearing sphere
Source: PLoS One. 2023 Sep 21;18(9):e0291719. doi: 10.1371/journal.pone.0291719 (PMC10513323; doi:10.1371/journal.pone.0291719)
Supplement: Attachment — Submitted filename: Response to Reviewers.docx [file pone.0291719.s002.docx]

Dear Reviewer/Editor:

Attached is this letter as a response to your valuable feedback. Thank you for providing useful suggestions and feedback during the review of our paper. Here are our detailed answers to your comments and questions.

Response to Reviewers：

a) About the ABAQUS model:

1. Thank you for your attention to our paper and for raising the question of “How was the load applied”. Here’s our more detailed explanation of these issues: We first set a reference point on the top plane of the pile, and then couple the reference point with the top plane of the pile in the "Interaction" module. In the "Load" module, we apply a point load to the selected reference point, then the point load can be coupled to the top plane of the pile. Due to the study of the ultimate bearing capacity of piles with ram-compacted bearing sphere in this article, the applied load is generally greater than the expected ultimate bearing capacity. Then, in the visualization post-processing part, the load on the pile body when the settlement is 60mm is extracted as its ultimate bearing capacity.

2. Thank you for your attention to our paper and for raising the question of “Please provide the full list of parameters”. Here’s our more detailed explanation of these issues: Because the friction angle of soil is less than 30°, and the dilation angle is generally taken as the friction angle $\varphi$ subtract 30°, so the system defaults to a dilation angle of 0.1; We do not specify the Abs Plastic Strain, ABAQUS believes that the cohesion remains unchanged at this time, which is an ideal elastic-plastic model. The static earth pressure K0 is generally taken as 1-sin$\varphi$, and the default value of ABAQUS is adopted for other parameters not mentioned.

|  | Dilation Angle | Deviatoric eccentricity | Meridional eccentricity | Abs Plastic Strain | K0 |
| --- | --- | --- | --- | --- | --- |
| Soil1 | 0.1 | Calculated default | 0.1 | 0 | 0.71 |
| Soil2 | 0.1 | Calculated default | 0.1 | 0 | 0.67 |
| Soil3 | 0.1 | Calculated default | 0.1 | 0 | 0.57 |

3. Thank you for your attention to our paper and for raising the question of “The authors must present references for the soil-pile friction angle values reported and its range”. Here's our more detailed explanation of these issues: We noticed that the value of the friction Angle of soil may not be considered comprehensively in the article, so we added two more references, the coefficient of soil is referred to the reference [24], and the parameter of pile is referred to the references [25] and [26]. We are very grateful for the editor’s suggestion. We will add the correct citation and range definition of soil pile friction angle values in the revised version to ensure the accuracy and reliability of the paper.

4. Thank you for your attention to our paper and for raising the question of “The authors must better explain the "multiple ODB import" method considered”. Here’s our more detailed explanation of these issues: In the modeling process, we first used the Geostatic option provided by ABAQUS to do ground stress balance, but we found that the result of this method was not converging, so we accepted the advice of our instructor and adopted the method of odb file import, but the overall displacement after only one import was too large, and the displacement was on the order of 10-3. It would have a certain impact on the test results, so we carried out repeated introduction to make the maximum displacement order of 10-5m. We believed that the displacement error at this time could be ignored and had little impact on the results, so multiple odb introduction method was adopted. The multiple ODB import method first establishes a “Static, General” analysis step, and then applies a force of -9.8N globally in the “Load” module for calculation. After the calculation is completed, return to the “Load” module and select the “Predefined field” command, the step selects “Initial”, and then select the “Stress” in mechanical. The specification selects “From output database file” to select the calculation file, fill in the analysis step and increment step required for calculation completion, and then proceed with the calculation. After completing the calculation, change the “Static, General” analysis step mentioned earlier to the “Geostatic” analysis step, then return to the “Predefined field” command to change the file to a new file and fill in the corresponding new analysis step and incremental step. At this point, the geostress balance is completed.

5. Thank you for your attention to our paper and for raising the question of “The authors must justify mesh sizes”. Here’s our more detailed explanation of these issues: The mesh size is measured in meters. We realize that in finite element analysis, the mesh size has an important impact on the accuracy and reliability of the results, and a larger mesh may lead to a low accuracy of the results, while a smaller mesh may increase the calculation cost. Therefore, we refer to the values in reference [30] and systematically change the mesh size, and compare the results under each size. The mesh size suitable for this paper is finally determined by observing the changing trend of the mesh size.

6. Thank you for your attention to our paper and for raising the question of “Did the authors perform any convergence study for the mesh sizes?”. Here’s our more detailed explanation of these issues: Our goal is to ensure that the simulation results are convergent and have sufficient accuracy. Since the calculation results are convergent, we did not conduct further research on their convergence. As I am a first-year graduate student, I plan to reflect this part of the research in the subsequent research, so I hope the editor can pay more attention to it and provide valuable experience for my future articles.

7. Thank you for your attention to our paper and for raising the question of “Which type of finite element was used?”. Here’s our more detailed explanation of these issues: This paper uses 3D solid simulation. Although our research is related to axial symmetry, we did not choose to conduct axisymmetric simulation in this study. This decision is based on our detailed analysis and evaluation of the issue. We believe that under current models and conditions, the use of axisymmetric simulation is not necessary and may not have a significant impact on our main research objectives. In addition, 3D solid simulation are more intuitive, so this paper uses 3D solid simulation instead of axisymmetric simulation.

b) About the paper:

1. Thank you for your attention to our paper and for raising the question of “Is it really important to consider side friction?”. Here’s our more detailed explanation of these issues: We believe that considering pile side friction is important. Generally speaking, the bearing capacity of pile is provided by pile side friction resistance and pile end resistance, even if the pile end bearing capacity is the main bearing mode, there will be pile side friction. From a practical point of view, there are also some drawbacks to considering side friction. One of the disadvantages is that side friction is difficult to measure directly, relying on experimental or empirical formulas for estimation. In addition, due to the heterogeneity and variability of the soil, the values of side friction may vary at different locations and depths, posing challenges for estimation and verification. Although the quality of the contact between the soil and the pile may vary with the heterogeneity during construction, even where the contact quality is poor, side friction can provide additional support by increasing the friction between the pile and the soil, thereby increasing the total load carrying capacity of the pile.

2. Thank you for your attention to our paper and for raising the question of “The authors would consider infinite elements on future works, but I am not sure this is really an issue to be dealt with”. Here’s our more detailed explanation of these issues: Infinite element is a special type of element that can extend the application of finite element analysis. They introduce an approximate infinity condition at the boundary, which allows better simulation of unbounded problems. This is very useful for problems with characteristics such as infinite fields, boundary free surfaces, etc. We mentioned in the paper that we would consider using infinite elements in future studies because although current model sizes may be large enough, we realize that conventional finite element meshes may encounter limitations when dealing with larger structures or domains. Therefore, in future research, the use of infinite elements is considered in order to solve such a potential problem. We understand the editors’ concern about the reasonableness of the size of the existing model. Our goal in this paper is to provide a workable approach and discuss its applicability and potential extensibility. Although the current model size may not require the use of infinite elements, given the possibility of larger scale problems, we want to express our thoughts on the direction of future research. However, considering Reviewer #2’s suggestion that “Conclusion and Recommendation can be combined. Some non-conclusive statements can be removed”, we decided to remove the prospect of establishing an infinite meta-model in the revised version and improve our conclusions and recommendations to ensure the simplicity of the paper.

3. Thank you for your attention to our paper and for raising the question of “The authors would consider infinite elements on future works, but I am not sure this is really an issue to be dealt with”. Here’s our more detailed explanation of these issues: The existence of side friction can effectively increase the bearing capacity of piles. Even where the contact quality is poor, side friction can provide additional support by increasing the friction between the pile and the soil, thereby increasing the total load carrying capacity of the pile. And the side friction is also very important to the lateral force resistance of pile. Even when calculating purely vertical load capacity, taking into account side friction can provide additional stability and resistance to side tipping. Considering the side friction can increase the safety factor in the bearing capacity calculation. By taking side friction into account, the behavior of soil-pile systems can be evaluated more comprehensively and some conservatism can be introduced into the design, thereby improving the safety of the structure. Therefore, although different soil-pile contact conditions vary, it is still important to consider the side friction for calculating the bearing capacity of piles. This helps to integrate the frictional characteristics between soil and pile, and improves the accuracy and reliability of the design.

4. Thank you for your attention to our paper and for raising the question of “Comparing the bearing capacities obtained by the Chinese Standard and the finite element simulation should be made in two scenarios”. Here’s our more detailed explanation of these issues: We acknowledge that comparing the bearing capacities obtained by the Chinese Standard and finite element simulations in two different scenarios, namely pure base contributions and base+friction contributions, is indeed necessary to fully evaluate the correctness of the standard’s intended purpose. To address this concern, we will consider the editor’s suggestion and include an additional analysis specifically focusing on the pure base contributions. This will allow us to provide a more comprehensive evaluation of the standard’s performance and its deviation from finite element simulations. We appreciate the editor’s valuable feedback and will incorporate this suggestion into our revised manuscript. Please let us know if you have any further comments or recommendations.

5. Thank you for your attention to our paper and for raising the question of “It is important to show how different values of the penalty friction impact the final bearing capacity”. Here’s our more detailed explanation of these issues: We believe that, as mentioned in formula (6), the value of penalty friction is related to the friction Angle of soil around the pile, and the property of soil around the pile has not changed in this paper, and we believe that the pile-soil contact is good. Therefore, we believe that the penalty friction between the pile and soil will remain unchanged during loading. Therefore, we did not reflect the research on how different values of the penalty friction impact the final bearing capacity in the paper.

6. Thank you for your attention to our paper and for raising the question of “It is important to show how different values of the penalty friction impact the final bearing capacity”. Here’s our more detailed explanation of these issues: Literature [3] and [13] are highly relevant to the research topic, and the authors are well-known and influential in this field. They have conducted comprehensive and in-depth literature research and used innovative methods, which are high-quality literature sources. Therefore, we will carefully study and evaluate the ideas and results presented in the existing literature [3] and [13], and will further deepen our discussion and analysis to ensure that they are different or more in-depth than those presented in the existing literature. We will look for new perspectives, provide new explanations, or extend existing theoretical frameworks to make our discussions unique and add them to the paper.

Response to Reviewers：

Reviewer #1:

（1）Thank you for your attention to our paper and for raising the question of “Different font sizes are used on page 12 and others, which should be avoided”. Here’s our more detailed explanation of these issues: We fully recognize the importance of maintaining a consistent font size throughout the paper, so we will carefully review and correct the font size differences between page 12 and other pages, and will use a uniform font size to maintain consistency throughout the paper. We are grateful to the reviewer for pointing this out, and we will correct this error in the revised version and ensure that the font size remains consistent throughout the paper.

（2）Thank you for your attention to our paper and for raising the question of “All the parameters used in the numerical modeling are assumed, which makes the obtained results not convincing and practical”. Here’s our more detailed explanation of these issues: In our study, we admit to using some parametric assumptions. These assumptions are made to simplify the problem and make the model workable. We try to choose the most reasonable parameter values, but are aware that these assumptions may have an impact on the accuracy of the results. We understand reviewers’ concerns about whether our results are validated or verified by field or lab results. In this study, we focused on numerical modeling and analysis and performed simulations based on known material parameters and boundary conditions. We did not directly use the field or lab results for verification, because the author is a first-year master student and has no time to conduct model tests for the time being. Moreover, due to the impact of the epidemic, the project of piles with ram-compacted bearing sphere has been completed early or no new project has been started, so it is difficult to obtain field or laboratory data. However, the author plans to start the model test at the end of August and prepare to reflect the work of this part in the subsequent research. I hope reviewers can pay more attention to it and provide valuable experience for my future articles.

Reviewer #2:

1：Thank you for your attention to our paper and for raising the question of “The proofreading by a native English speaker for the revised manuscript is recommended”. Here’s our more detailed explanation of these issues: We recognize the importance of proofreading in English for authors whose native language is not English. This ensures a standard of fluency and accuracy in grammar, spelling and sentence patterns. We adopted the reviewer's suggestion and asked my English teacher to proofread our revised manuscript. Although we did not ask an institution for calibration, we still further improved the language quality and readability of the paper. We are very grateful to the reviewers for this suggestion and we will ensure that the paper is proofread in English before submitting the revised version to ensure that the language expression of the paper is more precise and professional.

2：Thank you for your attention to our paper and for raising the question of “The abstract should be concise and can be combined”. Here’s our more detailed explanation of these issues: We recognize that abstracts should be characterized by brevity. Abstract is an important part of the paper, it should be able to accurately summarize the background, purpose, methods and main results of the research. At the suggestion of the reviewers, we will carefully examine the content of the abstract and consider whether it is possible to combine it to increase the simplicity of the abstract. We will remove unnecessary redundant information and ensure that the summary conveys the core content in a more compact manner. However, we also need to balance brevity with completeness of information. We will ensure that the abstract still contains enough key information so that readers can understand the main content of the study and the important results. By answering this question, we hope to be able to clarify our considerations regarding simplicity and consolidation of abstracts and to answer reviewers’ questions. We will further improve our abstract in the revised version to ensure a balance between brevity and information integrity.

3：Thank you for your attention to our paper and for raising the question of “Figure 4, what is the criterion to define the zone for the dry concrete, compacted filling, compacted soil and affected soil in the model?”. Here’s our more detailed explanation of these issues: We are very sorry for the reviewer’s doubts about the picture due to our negligence. We assumed that the ram-compacted bearing sphere area was homogeneous, so there was no division of the dry concrete area and other fillers in the carrier area. We put the corrected picture in the revised version of Figure 4. In Figure 4, we use specific criteria to define the different regions. According to the field sampling and laboratory model test mentioned in literature [27], we determined that the affected soil mass ranges from 2D under the ram-compacted bearing sphere (twice the diameter of the r ram-compacted bearing sphere) to 1D on the side of the ram-compacted bearing sphere (1 times the diameter of the ram-compacted bearing sphere), that is, its outer diameter is more than 3 times the diameter of the ram-compacted bearing sphere. Site construction may affect the compaction range of piles with ram-compacted bearing sphere, such as compaction energy, compaction frequency, etc. The author plans to start the lab test at the end of August, preparing to reflect the influence of site construction on the compaction range in the subsequent research. I hope reviewers can pay more attention to it and provide valuable experience for future articles.

4：Thank you for your attention to our paper and for raising the question of “the unit of the cohesion should be kPa not Kpa”. Here’s our more detailed explanation of these issues: We fully recognize that it is very important to maintain the correct units in scientific research to ensure the accuracy and reliability of the results, so we carefully check all the places in the paper that refer to the unit of cohesion and correct it from the wrong "Kpa" to the correct "kPa". We are grateful to the reviewer for pointing out this error, which we will correct in the revised version and use the correct unit "kPa" for cohesion.

5：Thank you for your attention to our paper and for raising the question of “It seems there is no experimental data for the verification of the numerical results”. Here’s our more detailed explanation of these issues: We did not directly use the field or lab results for verification, because the author is a first-year master student and has no time to conduct model tests for the time being. Moreover, due to the impact of the epidemic, the project of piles with ram-compacted bearing sphere has been completed early or no new project has been started, so it is difficult to obtain field or laboratory data. Although there is no experimental data to verify our numerical results, we still strive to verify the reliability and accuracy of the numerical simulation through reasonable parameter selection and other means. In addition, the author plans to start the lab test at the end of August, and is ready to reflect the work of this part in the subsequent research. I hope reviewers can pay more attention to it and provide valuable experience for future articles.

6：Thank you for your attention to our paper and for raising the question of “Figure 9: please use different legends rather than different colors to represent ZC1 to ZC6. Similar comments for Figures 10 and 11”. Here’s our more detailed explanation of these issues: Thank you very much for your review of our paper and valuable suggestions. We understand your concern, however, because of the amount of drawing data, using different legends in the graph can result in the graph being too dense and unsightly. Therefore, in this study, we chose to use a different color instead of a different legend to represent the graph. We believe that by using different colors to distinguish the data and providing sufficient explanation in the legend, the reader can still better understand the information presented in the chart. This visual presentation helps to avoid overcrowding the graphics while maintaining clarity and legibility. We value your input and will consider using different legend presentation methods in our future work. If you still have other suggestions or any other aspects of the diagram, we will be more than happy to listen and improve. Thank you again for your review and look forward to your further guidance and suggestions.

7：Thank you for your attention to our paper and for raising the question of “Conclusion and Recommendation can be combined. Some non-conclusive statements can be removed”. Here’s our more detailed explanation of these issues: We understand that combining conclusions and recommendations can make the paper more compact and clear. By combining the two sections, readers can more easily obtain a summary of the findings and further practical recommendations. We will carefully examine and evaluate non-deterministic statements in the paper and consider whether it is necessary to remove some of them. Removing these non-deterministic statements can enhance the certainty of the paper and make the conclusions and recommendations more actionable. However, we also need to balance brevity with completeness of information. We will ensure that all important and relevant information is still communicated when combining conclusions and recommendations and removing non-deterministic statements. By answering this question, we hope to be able to clarify our consideration of the merger of conclusions and recommendations and the removal of non-deterministic statements, and to answer the reviewer’s questions. We will further improve our conclusions and recommendations section in the revised version to ensure a balance between brevity and information integrity.

Thank you again for your review of our paper and your valuable suggestions. We sincerely appreciate your time and expertise and sincerely hope that our response meets your requirements and improves our research. If you have any further questions or comments, we will be happy to listen to them and modify them accordingly. We look forward to working closely with you to improve the quality of this paper.

Once again, express our gratitude and respect.

Sincere regards,

Author.
