# Supplementary material for: Finite element simulation study on vertical bearing characteristics of single pile with ram-compacted bearing sphere
Source: PLoS One. 2023 Sep 21;18(9):e0291719. doi: 10.1371/journal.pone.0291719 (PMC10513323; doi:10.1371/journal.pone.0291719)
Supplement: Attachment — Submitted filename: Response to Reviewer#2.docx [file pone.0291719.s003.docx]

Dear Reviewer#2,

Thank you for your prompt response and confirmation that the authors have adequately addressed most of the comments from the reviewer. We greatly appreciate your positive assessment and recognition of our efforts.

We have carefully revised the manuscript based on your valuable feedback and suggestions. Your encouragement and support are instrumental in improving the quality of our work. We believe the revised version is now ready for publication.

Once again, we would like to express our gratitude for your time and expertise in reviewing our manuscript. If there are any further steps or actions required from us, please do not hesitate to let us know.

Sincerely,

Author.
